# Combination of Modified Scarf Osteotomy and Metatarsal Shortening Offset Osteotomy for Rheumatoid Forefoot Deformity

**DOI:** 10.3390/ijerph181910473

**Published:** 2021-10-05

**Authors:** Yuki Etani, Makoto Hirao, Kosuke Ebina, Takaaki Noguchi, Gensuke Okamura, Akira Miyama, Hideki Tsuboi, Akihide Nampei, Shigeyoshi Tsuji, Hajime Owaki, Seiji Okada, Jun Hashimoto

**Affiliations:** 1 Department of Orthopaedic Surgery, Graduate School of Medicine, Osaka University, Suita 565-0871, Japan; y_etani@hotmail.co.jp (Y.E.); seokada@ort.med.osaka-u.ac.jp (S.O.); 2 Department of Musculoskeletal Regenerative Medicine, Graduate School of Medicine, Osaka University, Suita 565-0871, Japan; k-ebina@ort.med.osaka-u.ac.jp; 3 Department of Orthopaedic Surgery, National Hospital Organization, Osaka Minami Medical Center, Kawachinagano 586-8521, Japan; n-takaaki@hotmail.co.jp (T.N.); s.tsuji@ommc-hp.jp (S.T.); junha89@gmail.com (J.H.); 4 Department of Rheumatology, National Hospital Organization, Osaka Minami Medical Center, Kawachinagano 586-8521, Japan; 5 Department of Orthopaedic Surgery, Osaka Rosai Hospital, Sakai 591-8025, Japan; gensuke.okamura@gmail.com (G.O.); tsubo1155@gmail.com (H.T.); 6 Department of Orthopaedic Surgery, National Hospital Organization, Osaka Toneyama Medical Center, Toyonaka 560-8552, Japan; akira_m1984@yahoo.co.jp; 7 Nampei Orthopaedics and Rheumatology Clinic, Katsuragi 639-2162, Japan; ak-nampei@umin.net; 8 Department of Orthopaedic Surgery, Japan Community Health Care Organization (JCHO) Osaka Hospital, Osaka 553-0003, Japan; owaki-hajime@osaka.jcho.go.jp

**Keywords:** rheumatoid arthritis, forefoot deformity, modified scarf osteotomy, metatarsal shortening offset osteotomy, disease activity, intermetatarsal angle between the 2nd and 5th metatarsal bones (M2-M5A)

## Abstract

With the progress of medical treatment for rheumatoid arthritis (RA), several joint-preserving forefoot surgical procedures have been established and performed. In this situation, we have been choosing the combined surgery: modified scarf osteotomy for the great toe and metatarsal shortening offset osteotomy for the lesser toes in RA cases. A retrospective observational study of 53 RA patients (mean follow-up period: 4.6 years) who underwent the surgery was completed. RA foot ankle scores were assessed, using the Japanese Society for Surgery of the Foot (JSSF) standard rating system, and a self-administered foot evaluation questionnaire (SAFE-Q) was also checked to evaluate clinical outcomes. For radiological evaluations, deformity parameters were measured using radiographs of the feet with weight-bearing. JSSF hallux and lesser toes scores and the SAFE-Q score showed significant improvement in all indices. HVA, M1-M2A, M1-M5A, M2-M5A, and sesamoid position were significantly improved after surgery. At the final follow-up, the hallux valgus deformity had recurred in 4 feet (7.5%), and hallux varus deformity had developed in 8 feet (15.1%). No case of recurrent hallux valgus deformity required revision surgery. Recurrence of dorsal dislocation/subluxation of the lesser toe MTP joint was seen in 6 feet (11.3%) after surgery. A combination of modified scarf osteotomy for the great toe and modified metatarsal shortening offset osteotomy for the lesser toes is one of the novel surgical procedures for rheumatoid forefoot deformity. Preoperative disease activity of RA negatively affected the clinical score of the hallux. The spread of M2-M5A was a risk factor for resubluxation of the lesser toe MTP joint.

## 1. Introduction

In the advances in drug therapy for rheumatoid arthritis (RA), the concept for preserving the metatarsophalangeal (MTP) joint had been established when correcting forefoot deformities. We have been choosing the combination surgery of modified scarf osteotomy for the correction of hallux alignment [1,2,3] and metatarsal shortening offset osteotomy for the reduction of dislocated/subluxated metatarsophalangeal (MTP) joints in lesser toes [2,4]. These procedures were reported to be useful for the reconstruction of rheumatoid forefoot deformity, as well as other joint-preserving surgical procedures [5,6,7,8,9,10,11,12]. In this study, responses to a self-administered foot evaluation questionnaire (SAFE-Q) were also included to evaluate the combination surgery, and risk factors for recurrence of hallux valgus (HV) deformity and lesser toe MTP joint subluxation were also investigated. These evaluations, it was aimed to confirm the factors inducing the disadvantage for outcomes after the combined surgery. Furthermore, in this study, outcomes of this combined surgery were firstly reported with the comparison between preoperative and postoperative SAFE-Q scores.

## 2. Materials and Methods

A retrospective, observational study was completed for consecutive 60 RA feet which underwent combined forefoot surgery with modified scarf osteotomy and metatarsal shortening offset osteotomy for treatment of painful lesser toe MTP joint deformities (callosities) with HV deformity from July 2014 to May 2019. All cases had symptomatic moderate-to-severe forefoot deformity (hallux valgus angle (HVA) ≥ 25° with subluxation of the MTP joint with infectious or painful callosity in lesser toes). Conservative treatment had failed. The inclusion criteria were: (1) previously diagnosed as rheumatoid arthritis (RA) and prescribed medications; (2) a minimum follow-up duration of two years; and (3) availability of dorsoplantar weight-bearing radiographs of the feet that had been taken preoperatively, and at the time of the final follow-up. The exclusion criteria were foot treated with the combined surgery concomitant with other surgical treatment in another site of the foot. Taken together, within 60 feet, 53 feet were investigated. The duration of postoperative observation was between 2 and 7 years. This research was approved by the Institutional Ethics Review Board at the Osaka University Hospital (approval number: 14219). Patients’ demographics are shown in Table 1.

### 2.1. Surgery and Postoperative Procedure

#### 2.1.1. Modified Scarf Osteotomy (Great Toe)

Modified scarf osteotomy was performed as described previously [1,2,3]. After the osteotomy and correction, internal fixation was performed using AcuTwist screws (Acumed, Hillsboro, OR, USA). After that, the flap of the capsule and soft tissue was interposed into the newly formed first MTP joint and then sutured to the lateral wall of the capsule and dissected adductor hallucis tendon in all cases. The medial capsule was sutured after some shrinkage by the interposition of the 10-mm-wide flap into the first MTP joint. The Akin osteotomy was added if the first toe touched and pushed the second toe.

#### 2.1.2. Modified Metatarsal Shortening Offset Osteotomy (Lesser Toes)

Modified metatarsal shortening offset osteotomy was performed as described previously [2,4]. The extensor digitorum longus (EDL) and brevis (EDB) tendons were preserved. Synovectomy in MTP joints was performed as possibly, especially in toes with severe dislocation. After shortening osteotomy and offset correction (dorsally in second-forth toes and medially in the fifth toe) was performed (in the original procedure, all metatarsal heads were shifted to the medial side [13]), the osteotomy site was fixed with 1.2-mm Kirschner wires (K-wire) for 2 weeks. After the fixation with k-wires, rigid tip-bone transplantation at the offset correction site was also added to obtain rigid primary fixation. After removal of the K-wires, full weight-bearing was allowed with the fitting of an arch support.

**Figure 1:**
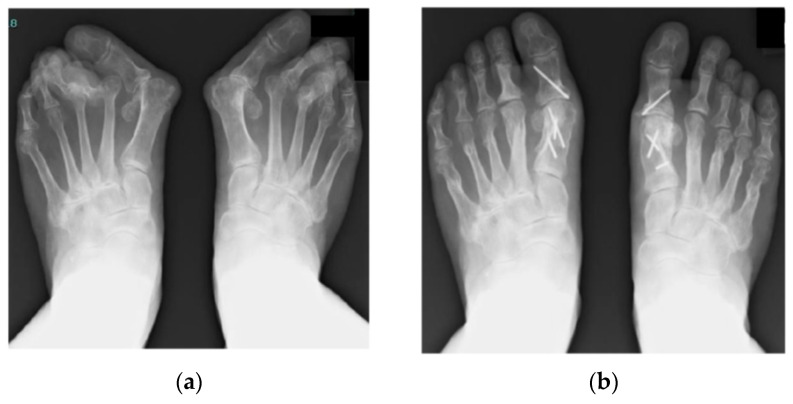
Representative case of a 75-year-old woman with a 40-year history of RA. (**a**) Preoperative radiograph with weight-bearing. Severe hallux valgus deformity, subluxation/dislocation of the lesser toe MTP joints, and joint erosion are seen. (**b**) Postoperative radiograph with weight-bearing 7 years after surgery. Combination surgery of modified scarf osteotomy and metatarsal shortening offset osteotomy has been performed. Akin osteotomy was also added to control the rotation and alignment. Painful callosity disappeared, and correction has been maintained 7 years after surgery. She can run at present.

Figure 1 shows a representative case that underwent the combination surgery of modified scarf osteotomy for the hallux and metatarsal shortening offset osteotomy for the lesser toes. Preoperative and postoperative (7 years after surgery) radiographs are shown.

### 2.2. Clinical Assessment

Preoperative and postoperative scores for both the hallux scale and lesser toe scale were obtained using the Japanese Society for Surgery of the Foot (JSSF) standard rating system [14,15]. Furthermore, a postoperative self-administered foot evaluation questionnaire (SAFE-Q) [16] was also completed preoperatively and at final follow-up. RA disease activity was evaluated using the disease activity score (DAS28-CRP) [17].

### 2.3. Radiographic Assessment

On radiographs of feet with weight-bearing, the HV angle (HVA), the intermetatarsal angle between the 1st and 2nd metatarsal bones (M1-M2A), the intermetatarsal angle between the 1st and 5th metatarsal bones (M1-M5A), and the intermetatarsal angle between the 2nd and 5th metatarsal bones (M2-M5A) were measured preoperatively and postoperatively. The sesamoid bone position was also evaluated (Hardy grade) [18]. These parameters were evaluated as described previously [4]. The preoperative tibio calcaneal (TC) angle was measured using a radiograph of the subtalar joint view (modified Cobey’s method [19]). Meary’s angle (inclination of the talus) was measured using a lateral view of the radiograph of the feet with weight-bearing [20]. On postoperative radiographs with weight-bearing, recurrence of MTP joint subluxation was evaluated. No joint space between the metatarsal head and phalanx bone or overlapping of the phalanx bone onto the metatarsal head was defined as recurrence of MTP joint dorsal subluxation/dislocation [4].

### 2.4. Statistical Analysis

All data are expressed as means and standard deviation (SD) or medians. Data were analyzed using the Statistical Package for the Social Sciences (SPSS) version 23.0 for Windows (IBM). The differences in the measured variables between the 2 groups were analyzed with the Wilcoxon signed-rank test or the Mann–Whitney U test, as appropriate.

## 3. Results

### 3.1. Clinical Outcomes

The mean follow-up duration was 4.6 years (range, 2 to 7 years). The mean JSSF RA hallux score improved significantly from 41.1 ± 10.8 points preoperatively to 88.4 ± 8.4 points at the final follow-up (Table 2). The mean JSSF lesser toe score improved significantly from 29.3 ± 11.2 points preoperatively to 85.2 ± 7.5 points at the final follow-up (Table 2). Each index of the JSSF score also improved significantly (Table 2). Preoperative and postoperative SAFE-Q scores are also shown in Table 2. Every index of the SAFE-Q score improved significantly after the combination of modified scarf osteotomy and metatarsal shortening offset osteotomy. The postoperative pain-related index, functioning index, and general health index were relatively good (score > 80). However, the score for the shoe-related index showed less improvement (score < 70) compared to the other indices.

### 3.2. Radiographic Outcomes

Generally, the HVA (from 41.7 ± 14.2 to 6.0 ± 9.4), M1-M2A (from 12.9 ± 4.6 to 5.1 ± 3.5), M1-M5A (from 32.4 ± 6.1 to 17.2 ± 5.4), and M2-M5A (from 19.5 ± 5.1 to 12.1 ± 4.3) decreased significantly at the time of final follow-up compared to preoperatively (Table 3). The sesamoid position also improved substantially and was maintained (Table 4). At the final follow-up, the HV deformity had recurred in 4 feet (7.5%), and hallux varus deformity had developed in 8 feet (15.1%) (Table 5). No case of recurrent HV deformity required revision surgery. Recurrence of dorsal dislocation/subluxation of the lesser toe MTP joint was seen in 6 feet (11.3%) after surgery (Table 5).

### 3.3. Relationship between Preoperative Radiographic Measurement or Disease Activity and Postoperative Outcomes

Multiple linear regression analysis with the postoperative HVA and JSSF hallux score as the dependent variables and the preoperative radiographic measurements and DAS28-CRP as independent variables was performed (Table 6). The results showed that there were no significant correlations between postoperative HVA and preoperative HVA, M1-M5A, Meary’s angle, and DAS28-CRP. Next, logistic regression analysis was performed to assess the predictors of postoperative recurrence of radiological HV (HVA > 20°) and hallux varus (HVA < 0°). Preoperative radiographic measurements and DAS28-CRP were used as independent variables, but no significant correlations were found in this analysis. On the other hand, preoperative DAS28-CRP showed a significant negative correlation with the postoperative JSSF score (β = −0.359, *p* = 0.04). Thus, higher disease activity was associated with a worse clinical outcome.

### 3.4. Risk Factors for Resubluxation of Lesser-Toe MTP Joints

Table 7 shows the results of the logistic regression analysis. When the risk factors for postoperative resubluxation of the lesser toe were analyzed, the preoperative M2-M5A showed a significant positive correlation (OR = 1.48, *p* = 0.04). On the other hand, preoperative HVA (OR = 0.90, *p* = 0.12) and preoperative DAS28-CRP (OR = 0.24, *p* = 0.33) did not show a significant correlation.

## 4. Discussion

The results of the present study, obtained after a mean follow-up duration of 4.6 years, demonstrate that joint-preserving surgery using the combination of modified scarf osteotomy and metatarsal shortening offset osteotomy improved clinical and radiographic outcomes in patients with RA. Although there was no preoperative parameter related to recurrence of HV, preoperative RA disease activity could affect clinical scores related to the hallux, as previously described [1]. Fortunately, hindfoot deformity, especially valgus hindfoot deformity, had no effect on recurrence, but, theoretically, valgus hindfoot had excessive loading on the medial part of the forefoot, and then a recurrence of HV deformity could easily occur [21,22]. Thus, we always should keep in mind the effect of hind-midfoot deformity on the forefoot. Comprehensive observation of the feet is important. Furthermore, whether Lapidus arthrodesis [23] should be added for increased M1-M2A cases should be further investigated in the future.

In the lesser toes, metatarsal shortening offset osteotomy was also useful for rheumatoid forefoot deformity. However, 11.3% of cases showed resubluxation of the lesser toe MTP joint, and the spread of M2-M5A was a risk factor for it (odds ratio: 1.48). Spread of M2-M5A means excessive loading on the lateral part of the fore-midfoot, leading to Lisfranc ligament disruption on the lateral side [24]. Excessive loading on the lateral part of the fore-midfoot might mean inversion and/or varus hindfoot deformity. Indeed, it was previously recognized that the varus hindfoot showed significantly more frequent recurrence of lesser toe MTP joint subluxation [4]. Therefore, comprehensive foot deformity should always be kept in mind. Because the total loading axis in the whole lower extremity might also have some effect on foot deformity and the loading pattern on the foot, investigation including these points has been performed in our institutions. It is also known that limitation of ankle dorsiflexion causes increased forefoot loading at the mid to terminal stance of gait [25]; therefore, existing bony ankyloses and/or arthrodesis of the ankle joint should have a key role in exacerbating recurrence of MTP joint dorsal dislocation/subluxation. Range of motion (ROM) exercises of the ankle joint and stretching exercises of the Achilles tendon should also be important to avoid recurrence. On the postoperative SAFE-Q evaluation, the shoe-related index showed a relatively low score (67.5) compared with other indices (82.5–86.5), which has been previously reported [4]. As a limitation, improvement of the score based on the "standing on toe" index was difficult after modified metatarsal shortening offset osteotomy [4,26]. Because these weak points were considered to be due to toe stiffness after surgery, we have been trying passive range of motion (ROM) exercise from the early period (2 weeks) after surgery [27]. To start early exercises, K-wires passing through the osteotomy site should be removed as early as possible. Then, rigid fixation at the offset osteotomy site is required. Transplantation of the tip-bone is considered important to obtain rigid fixation. Furthermore, as limitations, investigations in longer periods with a more increased number should also be done, because RA is a chronic and progressive disease. However, we believe that the combined surgery is one of the novel surgical procedures for rheumatoid forefoot deformity, as well as other novel combined joint-preserving forefoot surgeries [6,11,12] (Table 8).

## 5. Conclusions

The combination of modified scarf osteotomy for the great toe and modified metatarsal shortening offset osteotomy for the lesser toes is one of the novel surgical procedures for rheumatoid forefoot deformity. Preoperative disease activity of RA negatively affected the clinical score of the hallux. The spread of M2-M5A was a risk factor for resubluxation of the lesser toe MTP joint.

## Figures and Tables

**Table 1:** Characteristics of patients with rheumatoid arthritis (RA) and hallux valgus deformity (*N* = 53).

Characteristic	Results
Age * (y)	64.8 ± 11.5 (32 to 85)
Male:female (no.)	0:53
Disease duration * (y)	22.0 ± 12.0 (4 to 54)
Follow-up period * (y)	4.6 ± 1.9 (2 to 7)
Body mass index * (kg/m^2^)	21.5 ± 3.3 (17.5 to 29.4)
Steinbrocker stage (I, II, III, IV) (%)	4, 8, 24, 64
Steinbrocker class (I, II, III, IV) (%)	42, 47, 11, 0
DAS28-CRP *	2.8 ± 0.7 (1.18 to 3.99)
Biologics usage (%)	41.5
Biologics (no.)	TCZ: 11, ABT: 4, IFX: 2, ETN: 2, CTZ: 2, GLM: 1
MTX usage (%)	67.9
MTX dose * (mg/week)	6.6 ± 2.0 (4 to 10)
Prednisolone usage (%)	9.4
Prednisolone dose * (mg/day)	2.6 ± 1.2 (2 to 5)
Tibio-calcaneal angle *	3.3 ± 6.8 (−6 to 26)
Meary’s angle *	2.8 ± 9.7 (−32 to 37)
Ankylosis of the mid-foot (%)	22.6 (12/53)

* The data are presented as means and standard deviation with the range in parentheses. TCZ, tocilizumab; ABT, abatacept; IFX, infliximab; ETN, etanercept; CTZ, certolizumab pegol; GLM, golimumab; MTX, methotrexate; DAS28-CRP, disease activity score.

**Table 2:** Changes in the Japanese Society for Surgery of the Foot (JSSF) and postoperative self-administered foot evaluation questionnaire (SAFE-Q) scores.

Scores	Mean ± SD		*p*-Value
	Preoperative	Final Follow-Up	
JSSF hallux score			
Pain (40 points)	17.9 ± 7.4	38.1 ± 3.9	<0.001
Function (45 points)	20.9 ± 5.5	35.9 ± 5.2	<0.001
Alignment (15 points)	2.3 ± 3.6	14.3 ± 2.0	<0.001
Total	41.1 ± 10.8	88.4 ± 8.4	<0.001
JSSF lesser score			
Pain (40 points)	9.4 ± 10.0	38.3 ± 3.8	<0.001
Function (45 points)	18.9 ± 2.8	32.4 ± 4.0	<0.001
Alignment (15 points)	0.9 ± 2.5	14.5 ± 1.8	<0.001
Total	29.3 ± 11.2	85.2 ± 7.5	<0.001
SAFE-Q score			
Pain and pain-related (100)	44.4 ± 21.3	82.5 ± 15.8	<0.001
Physical functioning and daily living (100)	57.7 ± 22.5	82.9 ± 16.6	<0.001
Social functioning (100)	57.0 ± 30.2	84.6 ± 20.3	<0.001
General health and well-being (100)	53.2 ± 29.1	86.5 ± 18.9	<0.001
Shoe-related (100)	37.1 ± 23.6	67.5 ± 22.9	<0.001

**Table 3:** Changes in radiographic angle measurements in the forefoot.

Forefoot Deformity Parameters	Mean ± SD		*p*-Value
	Preoperative	Final Follow-Up	
HVA	41.7 ± 14.2	6.0 ± 9.4	<0.001
M1-M2A	12.9 ± 4.6	5.1 ± 3.5	<0.001
M1-M5A	32.4 ± 6.1	17.2 ± 5.4	<0.001
M2-M5A	19.5 ± 5.1	12.1 ± 4.3	<0.001

HVA, hallux valgus angle.

**Table 4:** Distributions of the positions of the medial sesamoid (Hardy grade).

Hardy Grade	No (%)	
	Preoperative	Final Follow-Up
1	2 (3.8%)	26 (49.1%)
2	3 (5.7%)	10 (18.9%)
3	2 (3.8%)	9 (17.0%)
4	2 (3.8%)	3 (5.7%)
5	8 (15.1%)	3 (5.7%)
6	18 (34.0%)	2 (3.8%)
7	18 (34.0%)	0 (0%)

**Table 5:** Postoperative complications after surgery.

Complications	% (No./Total No. of Feet)
Delayed wound-healing (hallux)	13.2 (7/53)
Deep/implant infection	0 (0/53)
Radiographic evidence of recurrence of hallux valgus	7.5 (4/53)
Radiographic appearance of hallux varus	15.1 (8/53)
Recurrence of callus	3.8 (2/53)
Intraoperative fracture at the site of osteotomy	0 (0/53)
Nonunion at the site of osteotomy (hallux)	0 (0/53)
Nonunion at the site of osteotomy (lesser toe)	0 (0/53)
Resubluxation of the lesser-toe metatarsophalangeal (MTP) joint	11.3 (6/53)
Ankylosis of the hallux MTP joint	0 (0/53)
Ankylosis of the lesser-toe MTP joint	7.5 (4/53)

**Table 6:** Correlation coefficients between postoperative HVA or the JSSF hallux score and preoperative disease activity/radiographic measurements.

Postoperative Outcomes (hallux)	Factors	β	95% CI	*p*-Value
Postoperative HVA vs.	Preoperative DAS28-CRP	0.214	−1.2 to 7.1	0.16
	Preoperative HVA	−0.158	−0.32 to 0.11	0.34
	Preoperative M1-M2A	−0.127	−0.94 to 0.41	0.44
	Preoperative Meary’s angle	0.111	−0.24 to 0.55	0.44
Postoperative JSSF hallux score vs.	Preoperative DAS28-CRP	−0.359	−7.7 to −1.1	0.01
	Preoperative HVA	0.269	0.0 to 0.32	0.05
	Preoperative M1-M2A	−0.195	−0.79 to 0.06	0.10

**Table 7:** Risk factors for resubluxation of the lesser-toe MTP joint.

Risk Factors (Candidate)	Odds Ratio	95% CI	*p*-Value
Preoperative DAS28-CRP	0.24	0.01 to 4.23	0.33
Preoperative HVA	0.90	0.79 to 1.03	0.12
Preoperative M2-M5A	1.48	1.02 to 2.14	0.04

**Table 8:** Comparisons between current study and other studies of combined joint-preserving surgery in RA cases.

Study	Procedure (Combination)	*N*	FU (y)	Clinical Score Preop	Clinical Score Final
Niki et al. [6]	Lapidus + proximal shortening oblique osteotomy	39	3.0	52.2 (JSSF RA)	89.6 (JSSF RA)
Bhavikatti et al. [11]	Scarf + Weil	66	4.3	39.8 (AOFAS)	88.7 (AOFAS)
Yano et al. [12]	Modified Mann + shortening oblique osteotomy	53	6.0	288.8 (500) (SAFE-Q)	386.8(500) (SAFE-Q )
Current	Modified scarf + shortening offset osteotomy	53	4.6	249.4 (500) (SAFE-Q) 41.1 (JSSF hallux) 29.3 (JSSF lesser)	404 (500) (SAFE-Q) 88.4 (JSSF hallux) 85.2 (JSSF lesser)

FU, follow-up period; AOFAS, American Orthopaedic Foot & Ankle Society score.

## Data Availability

Data are not available due to ethical restrictions.
